# Primary Lumbar Hernia, Review and Proposals for a Standardized Treatment

**DOI:** 10.3389/jaws.2023.11754

**Published:** 2023-12-06

**Authors:** Jeroen Heemskerk, Jeroen Willem Alfons Leijtens, Sebastiaan van Steensel

**Affiliations:** ^1^ Department of Surgery, Laurentius Hospital Roermond, Roermond, Netherlands; ^2^ Department of General Surgery, Erasmus Medical Centre Rotterdam, Rotterdam, Netherlands

**Keywords:** lumbar hernia, treatment, surgery, abdominal wall, intestinal obstruction

## Abstract

A lumbar abdominal wall hernia is a protrusion of intraperitoneal or extraperitoneal contents through a weakness in the posterior abdominal wall, usually through the superior or inferior lumbar triangle. Due to its rare occurrence, adequate knowledge of anatomy and methods for optimal diagnosis and treatment might be lacking with many surgeons. We believe a clear understanding of anatomy, a narrative review of the literature and a pragmatic proposal for a step-by-step approach for treatment will be helpful for physicians and surgeons confronted with this condition. We describe the anatomy of this condition and discuss the scarce literature on this topic concerning optimal diagnosis and treatment. Thereafter, we propose a step-by-step approach for a surgical technique supported by intraoperative images to treat this condition safely and prevent potential pitfalls. We believe this approach offers a technically easy way to perform effective reinforcement of the lumbar abdominal wall, offering a low recurrence rate and preventing important complications. After meticulously reading this manuscript and carefully following the suggested approach, any surgeon that is reasonably proficient in minimally invasive abdominal wall surgery (though likely not in lumbar hernia surgery), should be able to treat this condition safely and effectively. This manuscript cannot replace adequate training by an expert surgeon. However, we believe this condition occurs so infrequently that there is likely to be a lack of real experts. This manuscript could help guide the surgeon in understanding anatomy and performing better and safer surgery.

## Introduction

A lumbar abdominal wall hernia (hereafter called lumbar hernia) is a protrusion of intraperitoneal or extraperitoneal contents [1, 2] through a weakness or rupture in the posterior abdominal wall. The diagnosis should not be confused with a herniation of the intervertebral disc at the lumbar level, confusingly also often referred to as “lumbar hernia.” Lumbar hernias are considered rare [3]. The existence of lumbar hernia was first suggested by Barbette in the late 17th century and published by Garangeot in the 18th century. Most cases have been described in men [4]. Approximately 20% of these hernias are supposed to be congenital and might be associated with other anomalies such as hydrometrocolpos and anorectal malformations [5–9]. The vast majority are acquired either primarily due to increased intra-abdominal pressure exceeding local abdominal wall strength, or secondarily due to accidental [10–12] or operative trauma [13, 14].

The inferior triangle of Petit and the superior triangle of Grynfeltt-Lesshaft are described [15, 16] as the two anatomical areas in which 95% of the cases of lumbar herniation occur, with a slight tendency towards the superior lumbar triangle [17]. The other 5% are more diffuse and should probably be considered an entirely different entity, consisting of a cicatricial hernia in the lumbar region often accompanied by bulging of the abdominal wall due to denervation disregarding any anatomical landmarks. These hernias are not within the scope of the current manuscript.

**FIGURE 1:**
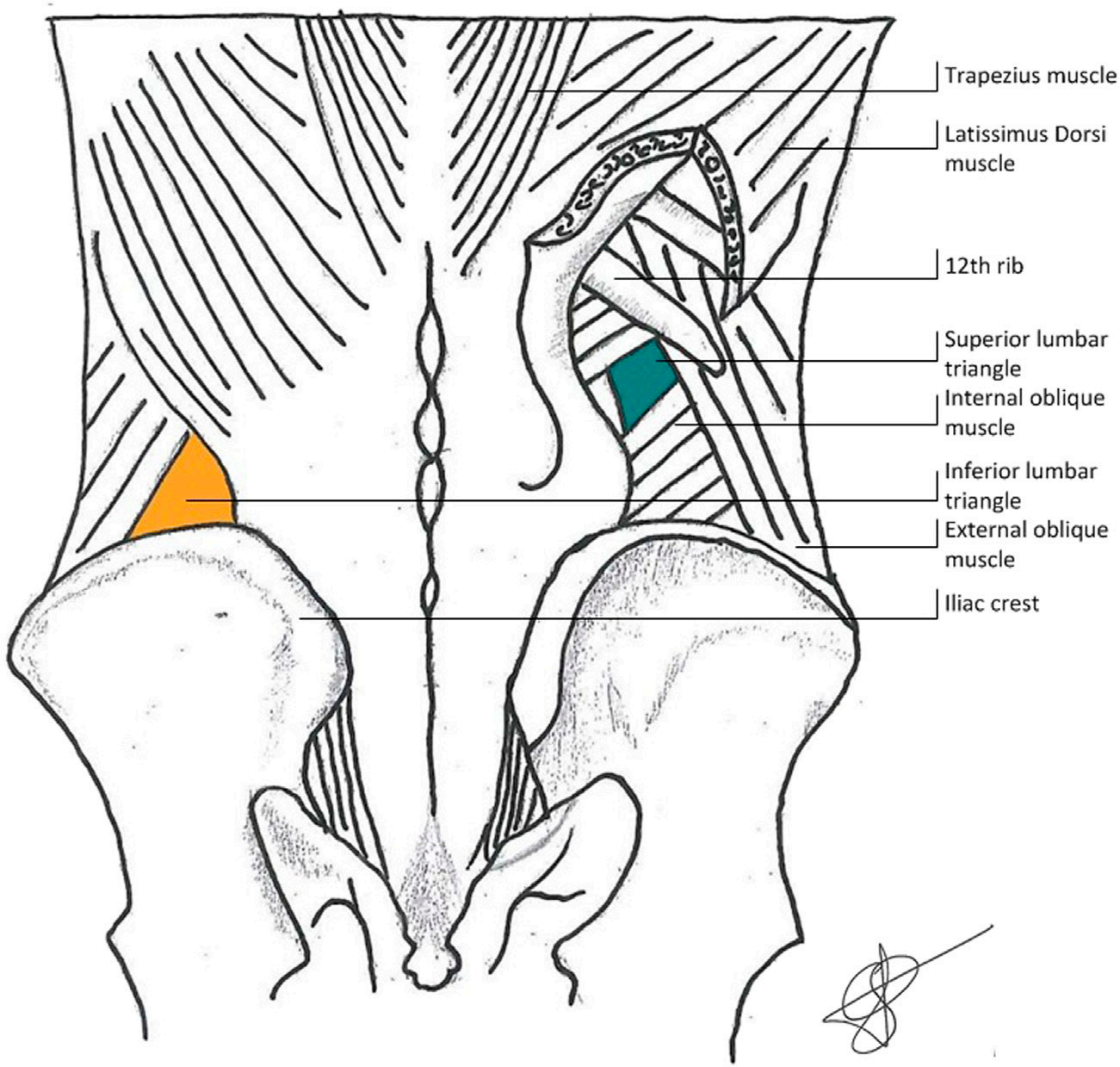
Anatomy of the lumbar abdominal wall.

Anatomically, the superior lumbar triangle is bordered superiorly by the posterior inferior serratus muscle and the 12th rib, laterally by the posterior border of the internal oblique muscle, and medially by the anterior border of the quadratus lumborum muscle. The inferior lumbar hernia is bordered medially by the lateral border of the latissimus dorsi muscle, inferiorly by the iliac crest, and laterally by the medial border of the external oblique muscle. Predisposing factors for the occurrence of primary lumbar hernia are anatomical factors (short, obese patients with relatively horizontal ribs and therefor a larger triangle) and general factors adding to elevated intra-abdominal pressure (pregnancy, obesity, ascites) or muscle wasting (neuromuscular diseases, aging, cachexia) [2, 18]. The anatomy can be seen in Figure 1 (courtesy of van Steensel, previously published in Hernia [19]).

Clinical presentation is a patient with a protrusion in the lumbar region that grows slowly and progressively and is valsalva positive. Pain or discomfort may occur [20–22]. Incarceration and strangulation do occur regularly in up to 30% of cases at primary presentation [4, 23–28], possibly due to the specific anatomical location or due to initial underdiagnosis. Since the exact risk of incarceration is unclear but certainly not negligible, operative treatment is advocated even in mildly symptomatic cases. Ultrasound can be used for diagnosis. However, the CT scan is now considered the gold standard because of its high sensitivity of 98%, lower interobserver variance, superiority in delineating the exact anatomy including fascial and muscular defects, its ability to specify herniated contents, and its superiority in determining the presence of muscle atrophy and bulging due to denervation [18, 29–31]. MRI may be useful [32], but superiority over CT scanning has not been shown at this time. It is advised to perform a CT scan routinely in order to allow for optimal assessment of anatomy and planning of surgery [2, 18, 19].

The goal of treatment is to decrease current symptoms and to prevent complications such as incarceration or obstruction [33, 34] by eliminating the defect and reconstructing an elastic but strong abdominal wall that is able to resist future stress. At the same time, unnecessary damage to the abdominal wall should be prevented. Nerves present in proximity to the superior lumbar triangle, such as the ilioinguinal, genitofemoral and lateral femoral cutaneous nerves are at risk of perioperative injury, which should be avoided. Failure to do so might result in neuralgic pain. Due to the rare appearance of this entity, large studies comparing different surgical strategies do not exist and a clear recommendation on which surgical technique to use cannot be given. Possibly, the anatomic variability as described by Loukas [15, 16] might be clinically relevant in predicting the risk of recurrence after surgery and in choosing the optimal surgical treatment strategy. Giant hernias and diffuse incisional hernias in the lumbar region are considered a different entity, often difficult to manage. Treatment of these hernias is often unsatisfactory [30], especially if local denervation results in bulging of the abdominal wall. Careful consideration should be given when deciding if surgical treatment should be offered at all to specific individual patients. If treatment is deemed necessary, it should be performed in an expert center for abdominal wall reconstruction, offering an individualized surgical strategy based on specific anatomical and medical conditions of the individual patient, possibly necessitating fixation of the mesh to bony structures [35].

Open surgical treatment has been performed for a long time [36, 37]. The use of a musculoaponeurotic [38] or de-epithelialized dermal [39] flap to cover the defect was described in 1907 but can lead to flap ischemia, hematoma, seroma, and high recurrence rates [38, 40]. Open exploration with primary closure of the defect, reconstruction of the resilient abdominal wall, and reinforcement by placement of a mesh was introduced in 1950 [41] and has proven to be an effective strategy [18]. However, an open surgical approach of the posterior abdominal wall can be demanding due to difficulties in optimally visualizing the external edges of the fascial defect, lack of sufficient resilient abdominal wall to perform sufficient tension-free primary reconstruction [38, 40], and difficulty in positioning and fixation of an adequately sized light-weighted mesh offering sufficient overlap, preferably in the extraperitoneal space. Some authors advocate a double mesh technique in an attempt to compensate for suboptimal overlap and fixation [42]. The presence of nerves and bony structures in close proximity to the defect further limits the opportunities for reconstruction and increases the risk for inadvertent damage, potentially leading to debilitating neuralgia. Transfascial sutures should potentially be avoided in order to prevent nerve entrapment resulting in debilitating pain, although evidence is not convincing [43]. In rare cases, fixation to bony structures can be used to improve strength [35, 44]. In the case of inferior lumbar hernia, securing the mesh using bone anchors, or by passing nonabsorbable sutures through a hole drilled in the iliac crest, might be advocated. In the case of superior lumbar hernia, a nonabsorbable suture might be tied around the 12th rib. The mainstay of treatment should however consist of offering sufficient overlap of an adequately placed and secured mesh.

More recent studies show sufficient evidence to support the advantage of laparoscopic repair, offering less postoperative pain and less analgesic consumption, thus resulting in faster convalescence and lower costs [4, 45–48]. Laparoscopy also offers superior assessment of visceral contents, minimizing the probability of inadvertent injury to internal structures and allowing optimal mesh placement of an adequately sized mesh. Laparoscopic treatment can be performed using either laparoscopic intraperitoneal onlay mesh (IPOM), Transabdominal Preperitoneal Plasty (TAPP), or Total Extraperitoneal Plasty (TEP). Due to the rare occurrence of lumbar hernia and a consequent lack of sufficiently powered, well conducted, Randomized Controlled Trials, evidence concerning optimal treatment is missing. In the absence of evidence, an extrapolation of the EHS recommendations for laparoscopic treatment of ventral abdominal wall hernia [49] can be made and general principals regarding hernia repair should be followed. In that case, the use of a mesh significantly reduces the number of recurrences [50]. Closure of the fascial defect is recommended when possible, resulting in less recurrence, seroma formation, and bulging [51, 52]. Intra-peritoneal onlay mesh may result in more short-term disadvantages such as increased postoperative pain and a longer hospital stay [53, 54] compared to extraperitoneal mesh placement. It might also lead to an increase in long-term disadvantages such as an increased risk of visceral damage and increased intraperitoneal adhesion formation, potentially leading to small bowel obstruction, mesh infection, or fistula formation [55]. Although hard evidence is missing to support the superiority of extraperitoneal mesh placement [56], several studies suggest a mild preference for extraperitoneal mesh placement in order to prevent intra-peritoneal adhesions and its complications. It is likely that a flat, lightweight, macroporous, non-resorbable mesh is the optimal material to augment the abdominal wall. An adequate mesh overlap seems imminent to prevent recurrences. The mesh area-to-defect ratio appears to be more important to minimize recurrence than a standardized mesh overlap length of 5 cm [57, 58]. However, as long as hard evidence and clear guidelines concerning the optimal mesh area-to-defect ratio in lumbar hernia repair are missing, a pragmatic suggestion might be to strive for an overlap of at least 5 cm.

Theoretically, a totally extraperitoneal approach might be advocated [59, 60] as the most feasible and safe operation. When performed by an experienced and skilled surgeon, TEP offers the opportunity to avoid the abdominal cavity, potentially offering less short-term risk of intra-abdominal visceral or vascular damage and fewer intraperitoneal adhesions, decreasing long-term risk of small bowel obstruction. A second advantage might be not requiring mesh fixation, diminishing the risk of inadvertent nerve damage due to misplacement of tackers or sutures, thus preventing neuralgic pain [61]. However, TEP might be considered technically more demanding, requiring a longer learning curve compared to TAPP surgery. This longer learning curve has previously been suggested in TEP vs. TAPP treatment for inguinal hernia surgery [62, 63]. A longer learning curve might influence the potential for inadvertent damage to nerves, muscles or visceral structures before reaching proficiency. Considering the rare occurrence of lumbar hernia, the opportunities for the surgeon and their surgical team to become fully proficient in the performance of totally extraperitoneal lumbar hernia repair seem limited. TAPP surgery has been described by many authors as an effective and safe procedure [64–66]. Using an IPOM or TAPP technique, adequate fixation of the mesh seems imminent and can be performed using either absorbable tackers, nonabsorbable tackers, sutures, or fibrin glue. There is no convincing evidence to indicate the superiority of one type of fixation over another [67–69].

## Materials and Methods

Due to the rare occurrence of this condition, surgeons with an extensive experience in surgical lumbar hernia repair are not likely to exist. We believe it might be useful to critically assess the sparse existing literature and suggest a relatively safe and easy surgical approach. TAPP requires limited specific expertise in lumbar hernia repair for a surgeon who is familiar with inguinal hernia TAPP repair. The anatomy can be well visualized and inadvertent visceral or nerve damage to nearby crucial structures is unlikely to occur. In lumbar hernia repair, as in larger studies concerning inguinal hernia repair, the theoretical advantages of TEP over TAPP repair preventing abdominal access have not shown major clinical relevancy and may not outweigh the disadvantages of the increased technical challenges. Therefore, we propose TAPP surgery as the first-choice technique in situations where the surgeon and surgical team are not extensively trained and experienced in TEP lumbar hernia surgery.

Step 1 After anesthesia induction, the patient is placed on a bean bag in lateral decubitus position with the affected side elevated around 60°. In our experience, a mild flexion of the table allows for an increase in working space. The patient is securely fastened to the table. We use one 12 mm camera port and two 5 mm working ports for left-sided procedure. The 12 mm port is placed in the upper left quadrant. Two 5 mm ports are placed subcostally and in the left flank.

**FIGURE 2:**
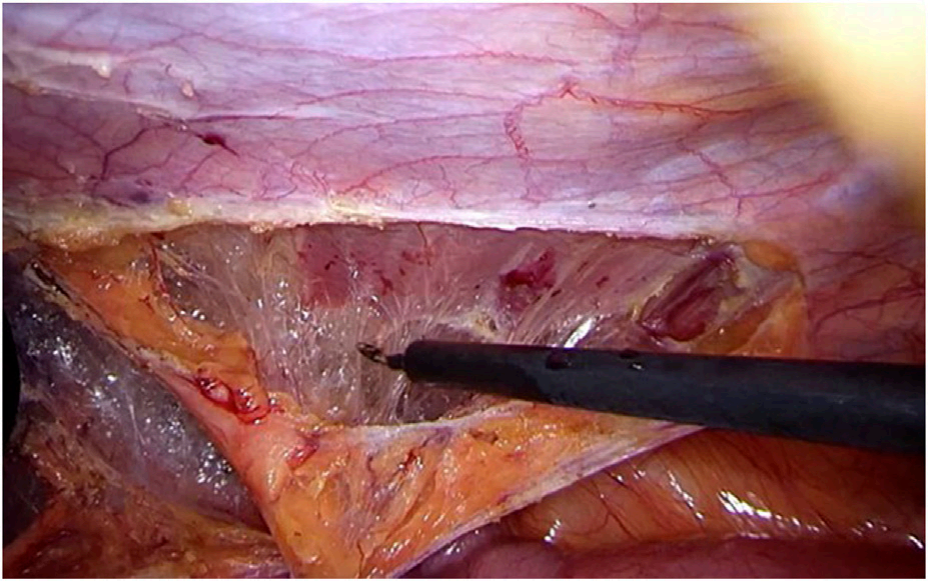
Clearing of the abdominal wall The colon, peritoneum, and adjacent extraperitoneal fat are mobilized from the abdominal wall musculature, adequately exposing the transverse fascia.

Step 2 Using sharp and diathermic dissection, the parietal peritoneum is incised just ventral to the splenic flexure. Care is taken to avoid intraperitoneal damage to spleen, greater omentum, or intestinal structures. Subsequently, the splenic flexure of the colon, parietal peritoneum, and adjacent extraperitoneal fat are mobilized from the abdominal wall musculature (Figure 2), adequately exposing the transverse fascia.

**FIGURE 3:**
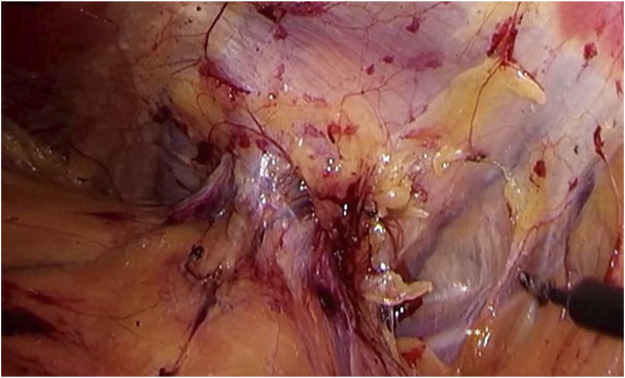
Identification of the iliohypogastric and ilioinguinal nerves. Extending dissection laterally and dorsally, the hernia port with protruding content can be visualized. The iliohypogastric (*) and ilioinguinal (**), running on the ventral border of the quadratus lumborum muscle just dorsal from the hernia port can be well visualized. More extensive dissection will also reveal the genitofemoral and cutaneous femoral lateral nerves.

Step 3 Extending the dissection laterally and dorsally, the hernia port with protruding content can be visualized. The iliohypogastric, ilioinguinal, genitofemoral, and cutaneous femoral lateral nerves running on the ventral border of the quadratus lumborum muscle just dorsal from the hernia port can well be visualized after adequate dissection (see Figure 3).

Step 4 Using gentle traction, the hernia contents can be reduced. Often, there is protrusion of extraperitoneal lipoma. It seems imminent to perform complete reduction of all hernia content in order to prevent persisting pain and bulging after surgery due to either seroma formation or the presence of a devascularized and still protruding lipoma through the defect in the abdominal wall.

**FIGURE 4:**
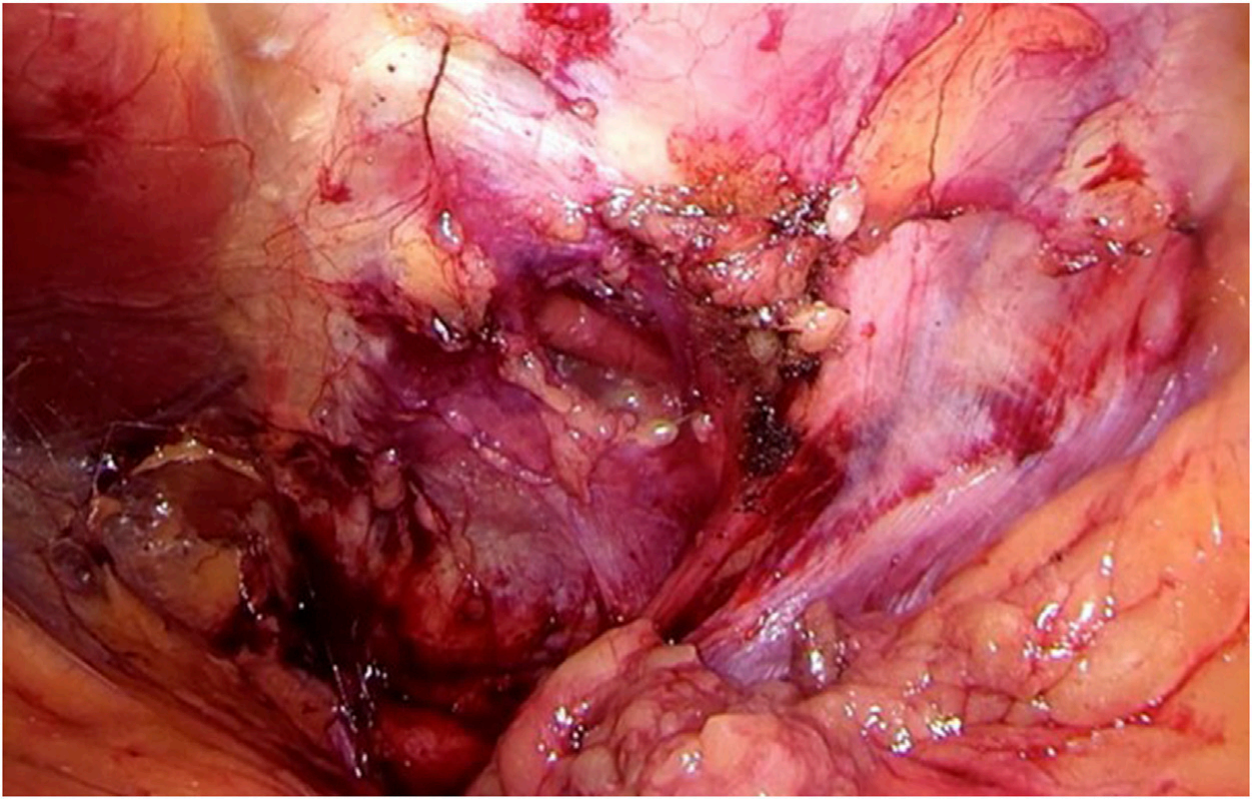
Once the contents are completely reduced, anatomy can be meticulously assessed and relation to relevant structures (such as nerves) can be well visualized. Careful interpretation of the course of the nerves seems imminent in preventing potential debilitating neuralgia by unintended nerve damage during port closure or mesh placement.

Once the contents are completely reduced, anatomy can be meticulously assessed and the relation to relevant structures (such as nerves) can be well visualized. Careful interpretation of the course of the nerves seems imminent in preventing potential debilitating neuralgia by unintended nerve damage during port closure or mesh placement (see Figure 4).

**FIGURE 5:**
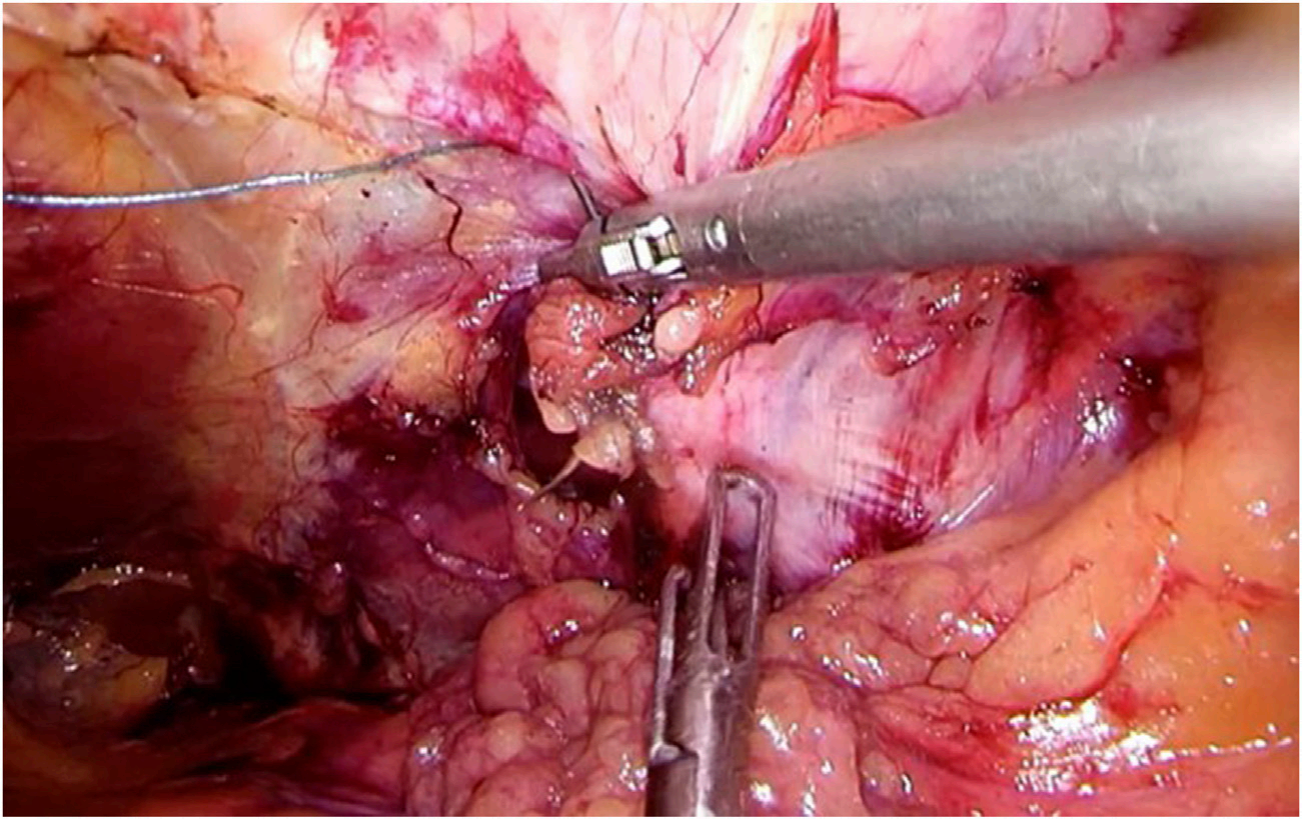
The hernia port rarely exceeds 4 cm and can usually be closed well, primarily using a slowly resorbable barbed suture. Care must be taken not to damage the nerves in close proximity to the hernia port.

Step 5 The hernia port rarely exceeds 4 cm and can usually be closed well, primarily using a slowly resorbable 3/0 barbed suture. Care must be taken not to damage the nerves in close proximity to the hernia port (see Figure 5).

**FIGURE 6:**
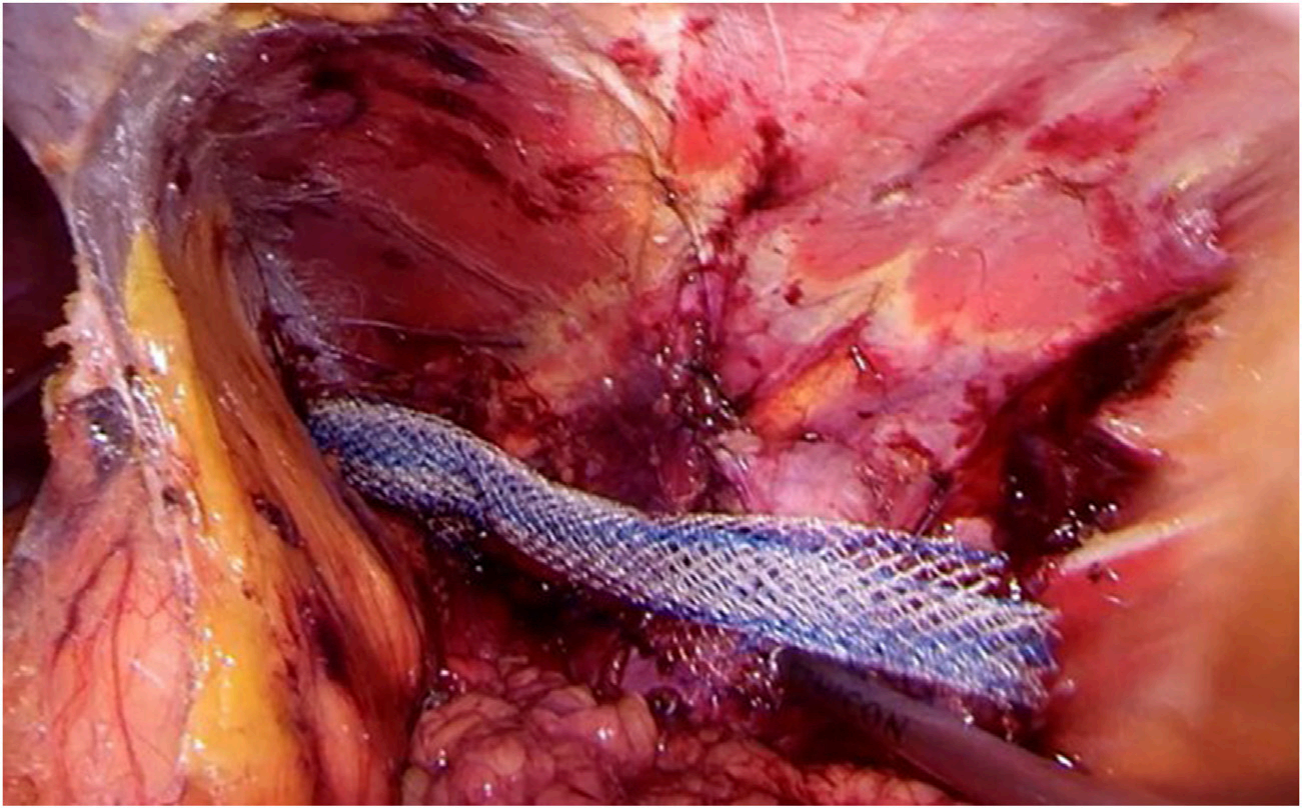
A flat, macroporous, and light-weighted mesh is rolled up and fixated with a temporary suture. Then, the mesh is inserted and the posterior border is secured using absorbable tacks, offering 5 cm overlap over the closed port.

Step 6 A flat, microporous, and light-weighted mesh is inserted, offering 5 cm overlap over the closed port. There is no hard evidence to suggest the superiority of one manufacturer over another. The mesh has been rolled and secured with a stay suture before insertion in order to allow easier handling. The dorsal border is fixated to the quadratus lumborum muscle at 5 cm distance from the port site using absorbable or nonabsorbable tacks. Care should be taken not to place tacks in close proximity to the nerves (Figure 6).

**FIGURE 7:**
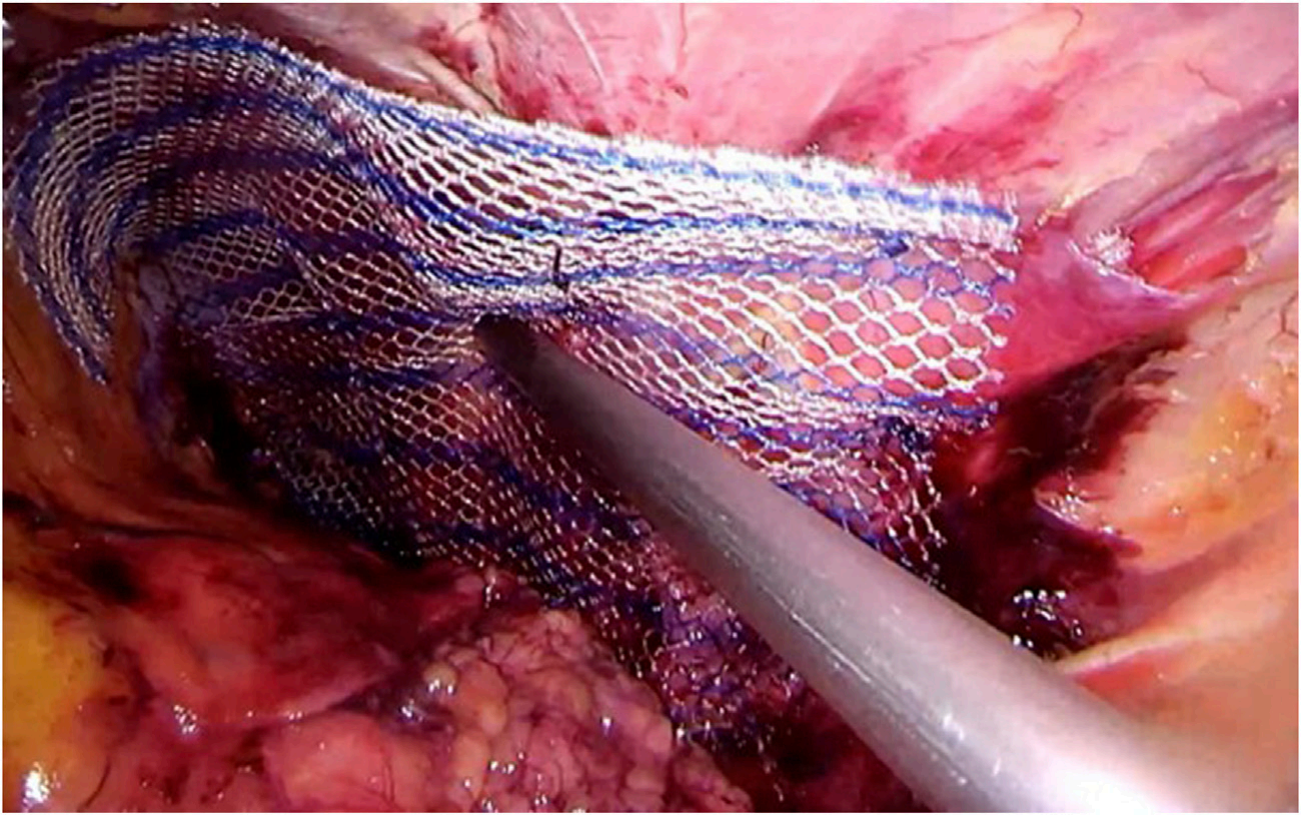
The temporary suture is cut, and the mesh can then be deployed and fixated using the same absorbable tacks to the serratus and anterior oblique muscles, offering the same 5 cm overlap.

Step 7 The temporary stay suture is cut, and the mesh can be easily deployed and fixated using the same tacks to the serratus and anterior oblique muscles (Figure 7).

**FIGURE 8:**
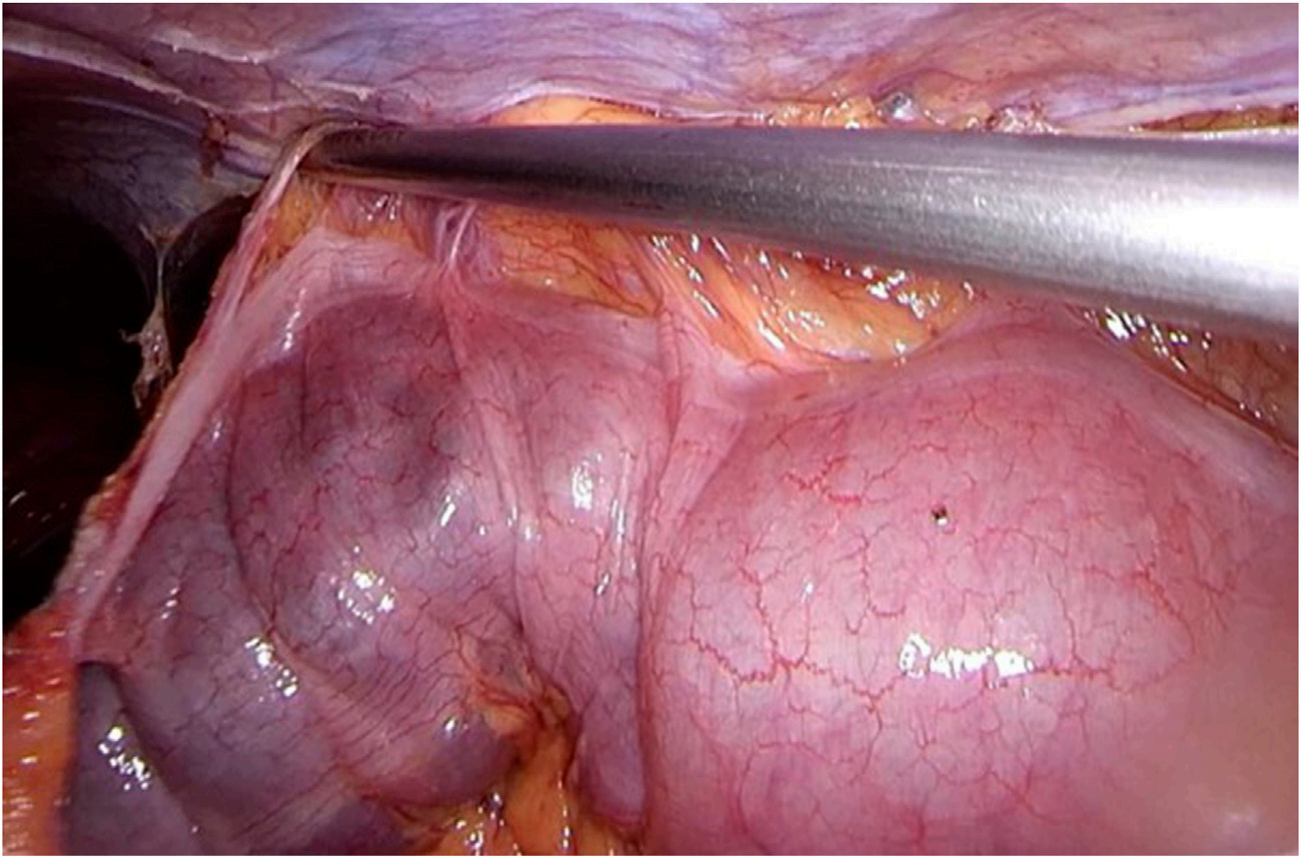
The peritoneum and splenic flexure are repositioned and fixated, making sure the mesh is well covered with peritoneum to prevent intra-abdominal complications. After desufflation, removal of trocars, and closure of the wounds, the operation comes to an end. The patient can be discharged the same day if postoperative pain is adequately treated using medication.

Step 8 The peritoneum and splenic flexure are repositioned and fixated, making sure the mesh is well covered with peritoneum to prevent intra-abdominal complications (see Figure 8). Desufflation, removal of trocars and closure of the wounds according to local protocol. The patient can be discharged the same day if postoperative pain is adequately treated using medication.

## Results

In our combined limited experience of only six patients (in four different hospitals), the use of a TAPP approach has proven easy to master, safe, and effective. All our cases were performed without complications such as neuralgia or hematoma, and we have not seen a recurrence yet. However, follow-up since the last case is only 5 months and our numbers are too low for an accurate recurrence rate.

## Discussion

Lumbar hernias are a rare entity and hard evidence on optimal diagnosis and treatment is lacking. Therefore, no one technique can convincingly be considered the gold standard. However, using the scarce literature available on this topic and adding information extrapolated from the literature on other hernias, a standardized, relatively safe, and easy strategy for surgical treatment might be proposed that can be performed by every adequately trained surgeon with significant expertise in hernia surgery.
